# Hepatitis E Outbreak on Cruise Ship

**DOI:** 10.3201/eid1511.091094

**Published:** 2009-11

**Authors:** Bengü Said, Samreen Ijaz, George Kafatos, Linda Booth, H. Lucy Thomas, Amanda Walsh, Mary Ramsay, Dilys Morgan, on behalf of the Hepatitis E Incident Investigation Team

**Affiliations:** Health Protection Agency Centre for Infections, London, UK (B. Said, S. Ijaz, G. Kafatos, A. Walsh, M. Ramsay, D. Morgan); Hampshire and Isle of Wight Health Protection Unit, Whitely, UK (L. Booth); North West London Health Protection Unit, London (H.L. Thomas); 1 Other team members are listed at the end of this article.

**Keywords:** hepatitis E virus, outbreaks, epidemiology, zoonoses, viruses, cruise ship, research

## Abstract

In 2008, acute hepatitis E infection was confirmed in 4 passengers returning to the United Kingdom after a world cruise. Epidemiologic investigation showed that of 789 persons who provided blood samples, 195 (25%) were seropositive, 33 (4%) had immunoglobulin [Ig] M levels consistent with recent acute infection (11 of these persons were symptomatic), and 162 (21%) had IgG only, consistent with past infection. Passenger mean age was 68 years. Most (426/789, 54%) passengers were female, yet most with acute infection (25/33, 76%) were male. Sequencing of RNA from 3 case-patients identified hepatitis E virus genotype 3, closely homologous to genotype 3 viruses from Europe. Significant association with acute infection was found for being male, drinking alcohol, and consuming shellfish while on board (odds ratio 4.27, 95% confidence interval 1.23–26.94, p = 0.019). This was probably a common-source foodborne outbreak.

## Abstract 2

The outbreak was probably foodborne.

In 1980, hepatitis E virus (HEV) was recognized as a cause of human disease (*1*,*2*). HEV infections can be asymptomatic or they can induce clinical hepatitis, which may be severe or life threatening, particularly for pregnant women. Other clinical manifestations associated with HEV infection have been reported. HEV is usually transmitted by the fecal–oral route and has an incubation period of 15–60 days (*3*). Four HEV genotypes that infect humans have been identified: genotype 1 is regularly found in HEV-endemic areas such as Africa and Asia; genotype 2 in Mexico and West Africa; genotype 3 in the United States, Europe, and Japan; and genotype 4 in Asia (*3*,*4*). Although HEV is increasingly recognized as a cause of hepatitis in industrialized countries (*5*,*6*), it is thought to be a relatively uncommon cause of viral hepatitis in the United Kingdom.

On March 27, 2008, the Southampton Port Health Authority informed the Health Protection Agency (HPA) of 4 elderly ship passengers with jaundice, who were returning from a world cruise. Because they had been fully vaccinated against hepatitis A, HEV was considered and subsequently identified as the probable causative agent. The ship had departed from Southampton, UK, on January 7 and returned on March 28, 2008. The ship had sequentially visited ports in Madeira, the Americas (South, Central, and North), the Caribbean region, Samoa, Tonga, New Zealand, Australia, Hong Kong, Thailand, Singapore, Malaysia, India, Egypt, Greece, and Spain before returning to the United Kingdom. Although the ship had only 1,800 passenger berths (cruise ship company data), the cumulative total of passengers during the cruise approached 3,000 because persons joined and left at different ports.

Because the outbreak of HEV was unusual, especially because it occurred on a cruise ship, and had potential public health implications, an epidemiologic investigation was undertaken. The investigation aimed to identify additional cases, help prevent future incidents by identifying possible risk factors for infection, describe the outbreak epidemiology, and further scientific understanding of the epidemiology and natural history of hepatitis E infection. The investigation was approved and commissioned by the HPA’s Hepatitis Programme Board. All participants had been passengers on board the cruise ship and volunteered and gave written informed consent. Ethics approval was not required.

## Methods

The investigation focused on all UK passengers who had been on the cruise at any point from January through March 2008. Contact addresses were provided by the cruise ship company, and 2,850 passengers were sent letters inviting them to participate in the investigation and explaining why. On the basis of when they were most likely to have been exposed (ascertained from the first 4 cases), participants were asked to go to their own doctors to give blood samples within 2 weeks (the time frame for detection of immunoglobulin [Ig] M). HPA provided sample kits with prepaid return packaging. Blood samples were tested for HEV antibodies (IgG and IgM) by using the Fortress Diagnostics ELISAs (Fortress Diagnostics Limited, Antrim, Northern Ireland) at the Virus Reference Department at the HPA Centre for Infections. Assays were run in accordance with the manufacturer’s instructions. The Fortress assays were chosen for this investigation because our validation exercises (data not shown) had demonstrated these assays to be more sensitive and specific than some other commercially available assays. Samples were screened for IgG, and those that were positive were then tested for IgM. The IgM-seropositive samples were further analyzed for HEV RNA, and those that were RNA positive were genotyped as previously described (*7*). Briefly, phylogenetic analysis of a 300-bp region of open reading frame 2 was conducted. The generated sequences were compared with genotype 3 sequences from the United Kingdom, Europe, and the United States and with genotype 1, 2, and 4 sequences retrieved from GenBank.

Participants returned self-completed questionnaires by mail. Detailed information was collected about demographic characteristics, potential risk factors and cofactors for disease (medical conditions, food and drink consumption, excursions, water exposure such as water activities in pools on the cruise ship and swimming in the sea while off the ship) and any relevant signs and symptoms. All HPA documents and files containing patient identifiable information were handled and stored in compliance with Caldicott guidance (*8*).

Participants were classified according to their serologic results as having had recent acute infection (serologically confirmed by HEV IgM and IgG), past infection (serologically confirmed by HEV IgG only and therefore unlikely to have been acquired during the cruise), or no infection (serologically negative for HEV IgG). Those with recent acute infection also provided further blood samples for liver function testing and confirmatory HEV antibody testing. Patients with acute cases were followed up by the local Health Protection Unit of the HPA. Liver function tests were performed by local services, and results were reported back to the HPA on a specific form. Blood samples for HEV testing were returned by mail as described above. A symptomatic hepatitis case was defined as recent acute infection in a patient with signs and symptoms compatible with HEV infection, e.g., jaundice and/or dark urine and pale feces. To ensure no false-positive results, additional testing was conducted on samples taken at least 1 month later from these IgM-seropositive participants.

We compared risk factors and exposure for those with recent acute infection with those for seronegative controls. Persons who had evidence of past infection were excluded because they had probably been immune during the study period. Single and multivariable logistic regression, using Stata statistical software, release 10.1 (StataCorp, College Station, TX, USA), was performed to identify the most likely cause of the outbreak and to estimate time and place of exposure. Specific exposures with estimated odds ratios (ORs) >1, p<0.2, and at least 50% of cases of recent acute HEV infection, were included in a multivariable model. The least significant factor was dropped from the model in a stepwise fashion until all remaining exposures exhibited a significant association: p<0.05 and OR >1.

After the multivariable model was finalized, we added each port visited, 1 at a time, to identify where participants may have been exposed to HEV. The interaction between food item and location was also considered by using a stricter selection criterion of significance level p<0.01.

## Results

Of the 2,850 passengers, >1,100 volunteered and 851 (30%) participated in the investigation (Figure). Blood samples and questionnaires were available from 659 participants. Of these 659, age range was 22–92 years old (mean age 68 years); >90% participants were 55–79 years of age. Of 789 participants who gave blood samples, 426 (54%) were female and 363 (46%) were male.

**Figure:**
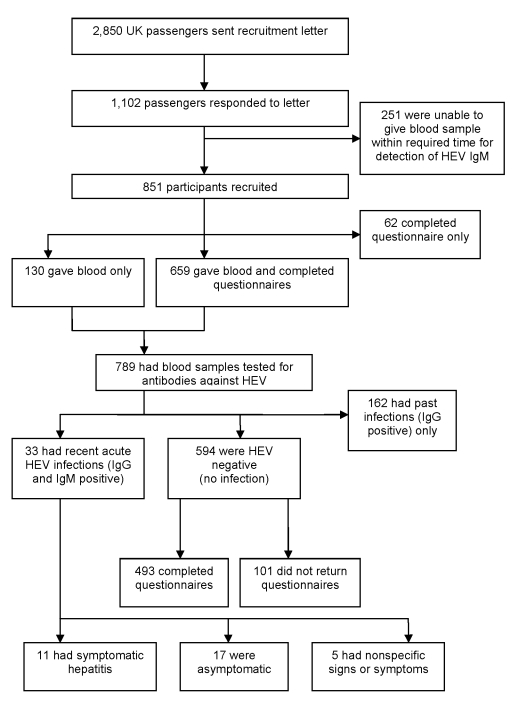
Recruitment of study participants from among UK passengers on world cruise with hepatitis E outbreak, 2008, and outcomes of epidemiologic investigation and clinical study. HEV, hepatitis E; Ig, immunoglobulin.

### Laboratory

Including the 4 case-patients identified on the cruise, 33 (4%) participants were classified as having had recent acute infections. A fall in IgM titer with a rise in IgG titer in the second sample collected 1 month later confirmed the acute infections. Another 162 (21%) were classified as having had past infection and 594 (75%) as having had no infection (Figure). Genotyping of RNA sequences obtained from 3 case-patients found genotype 3 virus with sequence homology close to that of other genotype 3 viruses reported throughout Europe.

### Statistical Analyses

Univariate analysis showed no statistical association between acute HEV infection and age group (Table 1). Infection was associated with gender; women were less likely than men to have been infected during the cruise. Some evidence indicated association with alcohol consumption; all nondrinkers were HEV negative, and those who drank alcohol (past or present) were more likely to have been infected. Alcohol was consumed by ≈84% of participants, 11% of whom exceeded the recommended weekly intake (defined as a maximum weekly intake of 21 units for men and 14 units for women). Medical conditions, including liver disease, did not appear to be significant risk factors.

**Table 1:** Univariate analysis of probability of having recent acute HEV infection, world cruise ship passengers, 2008, by participant characteristics*

Characteristic	HEV negative	HEV positive	OR	95% CI	p value
Age group, y (n = 526)					
<70	283	18	1.00	Baseline	
>70	210	15	1.12	0.55–2.28	0.749†
Gender (n = 526)					
M	224	25	1.00	Baseline	
F	269	8	0.31	0.14–0.69	0.002†
Past or present alcohol consumption (n = 523)					
No	49	0	1.00	Baseline	49
Yes	442	32	5.01	0.87–‡	0.061§
Amount of alcohol consumed (n = 415)					
Below recommended	349	23	1.00	Baseline	
Above recommended	37	6	2.46	0.97–6.26	0.058†

*HEV, hepatitis E virus; OR, odds ratio; CI, confidence interval. n values indicate number of responses received in each category. †χ^2^ test. ‡No upper limit. §Fisher exact test.

Odds of becoming infected were higher for passengers who had embarked from Southampton than for passengers who had joined the cruise at other ports (p<0.001). No evidence of association with recent HEV infection was found at any of the ports visited. Of those exposures ashore within the risk period, only excursions in Honolulu, Hawaii, USA (OR 2.22, 95% confidence interval [CI] 0.89–5.49, p = 0.086), and Pago Pago, Samoa (OR 3.26, 95% CI 1.41–7.53, p = 0.006), were significantly associated with infection.

Of the potential exposures on board, univariate analysis showed the following to be significantly associated with infection: unpasteurized cheese, paté, venison, and shellfish (Table 2). When shellfish were further differentiated (prawns, lobster, crab, mussels, scallops), the association appeared to be significant for lobster and crab; however, few HEV-positive participants said that they had eaten lobster (n = 9) or crab (n = 4). The final multivariable model (Table 3) showed the following to be associated with HEV infection: being male, drinking alcohol, and eating shellfish while on board.

**Table 2:** Univariate analysis of probability of having recent acute HEV infection, world cruise ship passengers, 2008, by potential exposures on board ship*

Exposure	HEV negative	HEV positive	OR	95% CI	p value
Ate shellfish (n = 499)					
No	113	2	1.00	Baseline	
Yes	356	28	4.44	1.17–16.83	0.025†
Ate lobster (n = 319)					
No	263	9	1.00	Baseline	
Yes	38	9	6.92	2.89–16.58	<0.001‡
Ate crab (n = 320)					
No	283	14	1.00	Baseline	
Yes	19	4	4.26	1.39–13.02	0.011‡
Ate shellfish in restaurant A (n = 263)					
No	187	7	1.00	Baseline	
Yes	63	6	2.54	0.85–7.61	0.094‡
Ate bacon (n = 497)					
No	75	1	1.00	Baseline	
Yes	392	29	5.55	0.93–33.25	0.067†
Ate cured pork (n = 454)					
No	272	14	1.00	Baseline	
Yes	154	14	1.77	0.83–3.77	0.141‡
Ate paté (n = 434)					
No	260	9	1.00	Baseline	
Yes	151	14	2.68	1.16–6.16	0.020†
Ate eggs (n = 499)					
No	39	0	1.00	Baseline	
Yes	430	30		Not estimable	0.155‡
Ate unpasteurized cheese (n = 465)					
No	194	7	1.00	Baseline	
Yes	240	24	2.77	1.21–6.37	0.016†
Ate venison (n = 451)					
No	326	17	1.00	Baseline	
Yes	96	12	2.40	1.13–5.10	0.023†
Swam in pool C (n = 494)					
No	336	16	1.00	Baseline	
Yes	130	12	1.94	0.9–4.16	0.089†
Participated in any water activities (n = 493)					
No	210	8	1.00	Baseline	
Yes	254	21	2.17	0.96–4.92	0.063†

*HEV, hepatitis E virus; CI, confidence interval. n values indicate number of responses received in each category. †χ^2^ test. ‡Fisher exact test.

**Table 3:** Multivariable analysis model of probability of having recent acute HEV infection, world cruise ship, 2008*

Exposure (n = 490)	OR	Profile likelihood, 95% CI	p value
Gender			
F	1.00	Baseline	
M	2.38	1.07–5.68	0.033
Age group			
<70	1.00	Baseline	
>70	0.96	0.97–1.08	0.384
Medication taken			
No	1.00	Baseline	
Yes	1.65	0.68–4.62	0.282
Any past or present alcohol consumption			
No	1.00	Baseline	
Yes		Not estimable	0.033
Shellfish consumed on board			
No	1.00	Baseline	
Yes	4.27	1.23–26.94	0.019

*HEV, hepatitis E virus; OR, odds ratio; CI, confidence interval.

### Recent Acute Infections

Of the 33 participants who had had recent acute infections, 25 (76%) were men 57–87 years of age (mean 68 years), and most (76%) were taking medication. All had drunk alcohol, 7 (21%) of whom had exceeded the recommended weekly units. Only 11 had symptoms compatible with hepatitis; 22 (67%) had either no symptoms or nonspecific symptoms of a cold (Figure). Age, gender distribution, and the proportion receiving medication were similar among those who were symptomatic (8 [73%] were male, average age 68 years; 8 [73%] were taking medication) or asymptomatic (13 [76%] were male, average age 69 years; 12 [71%] were taking medication). However, 4 (36%) symptomatic participants had consumed excess alcohol compared with only 1 (6%) asymptomatic participant.

Symptom onset was during March 6–24 (mode March 8, median March 12). Of the 11 symptomatic passengers, 5 had visited the ship’s doctor 3–5 days after symptom onset, 3 had been hospitalized, and 6 reported having been sick for 6–21 days (median 12 days).

Of the 11 (33%) participants who met the case definition for symptomatic hepatitis, all had loss of appetite, malaise, dark urine, and nausea. Other signs were jaundice and vomiting (n = 7); abdominal pain or discomfort or pale stools (n = 5); and headache, weakness, shakiness, joint pain, rash, or depression. Weeks later, a second phase of illness was reported by 2 participants; both experienced abdominal pain or discomfort, and 1 also had dark urine and lethargy. Blood test results for bilirubin, alanine aminotransferase, alkaline phosphatase, and albumin were available for 10 of 11 participants with symptomatic hepatitis. Levels >2× the expected maximum were found for bilirubin (n = 6), alanine aminotransferase (n = 5), and alkaline phosphatase (n = 2). Aspartate aminotransferase levels were reported for only 3 participants with symptomatic hepatitis and were elevated for 2. Subsequent testing for 2 case-patients showed that liver function had returned to within reference limits. Liver function test results were also reported for 14 of 22 participants who did not have symptomatic hepatitis; none were appreciably elevated. Blood samples from this group were taken later than for those with symptomatic hepatitis, so the possibility of abnormal liver function in the earlier phase of infection cannot be excluded.

### Exposure Period and Potential Source of Infection

Our analysis suggests that passengers were at higher risk for HEV infection if they were on the cruise from January 7, when the ship left Southampton, until February 14, when it arrived in Sydney. Most of those with recent acute HEV infection had embarked (January 7) and disembarked (March 28) in Southampton. However, 6 passengers, who were later seropositive, disembarked in New Zealand or Australia and 1 embarked in San Francisco and disembarked in Hong Kong. These dates indicate that the second leg of the cruise, from San Francisco (January 26) to Auckland (February 11), was the likely exposure period and suggest that the outbreak incubation period was 25–40 days.

A symptomatic case-patient who had late-onset disease had shared a cabin with a case-patient who had early-onset disease; the late-onset disease was potentially a secondary infection. Excluding this possible secondary case from repeat statistical analyses did not affect our results.

Information about onshore activity during this likely exposure period, including organized excursions, was available for 32 of 33 participants who had had recent acute infections. All 32 had gone ashore in Honolulu, Pago Pago, and Auckland. However, no common activities or excursions were noted, and most did not consume any food while ashore, except in Auckland. A variety of foods were consumed while ashore, and no common food was identified. Overall, analysis of excursions and food and drink consumed while ashore during the second leg of the cruise found no evidence that the infection was acquired while ashore.

Association between shellfish consumption while on board and recent HEV infection was further investigated. All seafood had been frozen; most was put on board in Southampton, but some was sent later from the United Kingdom or purchased in Australia. Lists of seafood used on board during the cruise were provided by the cruise ship company. Seafood was served on 34 of 38 days between Southampton and Sydney. Prawns and mixed seafood (mixture of shrimp or small prawns, salmon, cod, mussels, hake, and squid) were served most frequently. Implicating any particular shellfish was difficult because crab, lobster, mussels, scallops, shrimp, prawns and mixed seafood were all served at least 1 time during the suspect period of travel between San Francisco and Auckland.

## Discussion

This hepatitis E outbreak among passengers during a 3-month world cruise was reported at the end of the cruise, after passengers had already disembarked and returned home. Nevertheless, the fact that approximately one third of eligible passengers were able to give blood samples within a short time enabled detection of an acute antibody response. The testing algorithm was adopted on the basis of the onset dates of the first 4 cases and the expected time delay between contacting the passengers and actually receiving samples in the laboratory for testing. It was therefore thought to be unlikely that screening for HEV RNA would provide a useful marker of recent acute infection.

One study limitation was that participants were self-selected and may not therefore represent a random sample of passengers on the cruise. Also, the time between the cruise and completion of questionnaires may have made recalling foods consumed during the cruise difficult. However, most returned questionnaires were completed comprehensively, leaving no reason to suspect differential recall between case-patients and others.

Evidence of recent acute hepatitis E infection was found for 33 participants. The evidence of past infections for 162 (21%) is consistent with hepatitis E seropositive rates of ≈25% of UK residents >55 years of age (*9*). Only 11 participants with acute infection reported illness compatible with hepatitis; 22 (two thirds) were asymptomatic or had unrelated symptoms. This investigation provided a unique opportunity to diagnose asymptomatic infection in an exposed group and found a much higher asymptomatic rate than previously reported (≈3%–4%) (*3*,*10*). Elevated liver function test results appeared to be associated with symptomatic cases; however, because those without apparent clinical signs were tested a longer time after exposure, their liver function could have returned to within reference limits.

HEV RNA was detected in only 3 case-patients, suggesting that RNA had cleared by the time most samples were tested. Virus RNA sequences were identical and belonged to genotype 3, suggesting a common-source outbreak. Genotype 3 is the main type of HEV found in industrialized counties, including the United Kingdom. Although it has also been reported in North America, Southeast Asia, Australia, and New Zealand (*4*), the genotype 3 virus found in this outbreak had close sequence homology with genotype 3 strains reported in Europe.

The evidence implicates the second leg of the cruise (January 26–February 11). Although infection could have been acquired while ashore, the epidemiologic investigation suggests that exposure occurred while on board the ship. The estimated incubation period for this outbreak, 25–40 days, is shorter than but within the range of the reported incubation period for hepatitis E (15–60 days) (*3*).

The 3 associated risk factors (gender, age, shellfish consumption) remained significant in multivariate analysis. First, male passengers were more likely to have been infected than female passengers; 76% of recent acute infections were in men. A marked excess of indigenously acquired HEV cases in middle-age and elderly men has been reported in England, Wales (*5*), and other European countries (*10*–*12*). Our study suggests that this observed excess is not caused by ascertainment bias because men are at higher risk for disease, but rather it appears to be a genuine difference in exposure. Second, alcohol consumption was associated with recent infection. Although excess alcohol consumption could compromise hepatic function and predispose to symptomatic hepatitis E infection, as suggested by our study, alcohol consumption is probably not causally linked to exposure. The association may indicate a propensity for risk behavior that could not be controlled for in the analysis. Third, consumption of shellfish on board the ship was strongly associated with HEV infection. Further analysis did not implicate a particular type of shellfish, and cross-contamination of shellfish from a single vehicle is possible, but because the virus is waterborne and has been shown to contaminate shellfish (*13*–*15*), this association is biologically plausible. Other studies have shown that HEV can be foodborne, and illness has been linked to consumption of undercooked or raw meat (*16*–*18*).

Many of the 33 recent acute HEV infections, predominantly in men who drank alcohol, were asymptomatic and would otherwise have gone undiagnosed. We found no evidence of continuing transmission, other than 1 potential secondary case, or of any breaches of public health standards on board the ship. However, the analytical study and supporting genotype findings challenged the initial assumption that the outbreak was due to infection acquired while ashore. This investigation suggests that shellfish, which are known to be a common source of other viral infections, are a potential source of HEV infection in Europe.
